# Social Consequences of Infertility on Families in Iran

**DOI:** 10.5539/gjhs.v8n5p89

**Published:** 2015-08-31

**Authors:** Mohammad Amiri, Ahmad Khosravi, Reza Chaman, Zakieh Sadeghi, Mehdi Raei, Mohammad Ali Jahanitiji, Fardin Mehrabian

**Affiliations:** 1 Department of Public Health, School of Public Health, Shahroud University of Medical Sciences, Shahroud, Iran; 2 Center for Health Related social and Behavioral Sciences Research, Shahroud University of Medical Sciences, Shahroud, Iran; 3 Department of Community Medicine, School of Medicine, Yasuj University of Medical Sciences, Yasuj, Iran; 4 Analytical Chemistry, Shahroud, Iran; 5 School of Medicine, Qom University of Medical Sciences, Qom, Iran; 6 Department of Medicine, School of Medicine, Babol University of Medical Sciences. Babol, Iran; 7 School of Health, Guilan University of Medical Sciences, Rasht, Iran

**Keywords:** infertility, fertility, Consequences of Infertility, family, divorce

## Abstract

**Background and Objective:**

Social reactions to infertility are one of the concerns infertile people. This study aimed to investigate the social consequences of infertility among urban and rural population of Shahroud in northeast of Iran.

**Method:**

This study is a comparative study that was conducted in 2013. In this study, 1,528 women (511 infertile and 1017 fertile ones) were randomly selected. The 36-item questionnaire included 18 items about women’s attitude towards infertility and 18 questions about the consequences of infertility was used. Data were analyzed using chi-square test, one-way analysis of variance and t test.

**Findings:**

The prevalence of infertility in rural areas was estimated to be 2.23 percent. 42.2% of the participants were living the city (n= 645) and 57.8 % were living in the village (n=883). 49.2% of the participants had education below high school diploma (n=751), 31.7% had high school diploma (n=484) and 19.2% had university degrees (n=293). 51.9% of the people referred to the infertility problem among distant relatives, 24.9% referred to infertility among the close relatives and 9% reported the infertility among their family members. The mean score of attitude of the fertile was 56.6±7.0 and that of the infertile was 56.8± 6.6 and there was no statistically significant difference between the two groups (P>0.05). There was a significant association between fertility status and encouraging divorce, encouraging remarriage and encouraging adoption (P=0.001).

**Conclusion:**

Infertility causes a negative attitude toward infertile people. But the interference of others leads to further encouragement of divorce and remarriage among the infertile people.

## 1. Introduction

Despite various changes in attitudes toward sexuality in recent centuries, fertility has still remained important in the human mind and one of the factors which strengthens marital life is the existence of a child (Bahrami et al., 2010). Fertility or ability to have children is the success of reproduction and the beginning of a rebirth for couples, and its opposite, infertility through making reproductive disorders and as a phenomenon which sometimes is curable and sometimes incorrigible has always brought about various unintended consequences for the couples (Shahnooshi & Karimi, 2010). Infertility is one of the most important life crises and compared with stressful life events, after the death of the mother, father and wife infidelity is in the fourth place (Behjati Ardakani et al., 2010). Infertility is a process that lays influence on body, occupation, personality and mentality and adversely affects the feelings of the individual (Faal Kalkhoran, Bahrami, Farrokhi, Zeraati, & Tarahomi, 2011; Kormi Nouri, 2000). Some studies show that depression, stress, low self-confidence and sexual dissatisfaction are among the psychological consequences of infertility (Kormi Nouri, 2000). Infertility is defined as the inability to conceive after one year of regular sexual activity without the use of contraceptive methods (Fooladi, Danesh, Kashfi, Khani, & Mohammadpor, 2006; Marci et al., 2012; Monga, Alexandrescu, Katz, Stein, & Ganiats, 2004; Oskay, Beji, & Serdaroglu, 2010; Rascanu & Vladica, 2012; Shindel, Nelson, Naughton, Ohebshalom, & Mulhall, 2008; Valsangkar, Bodhare, Bele, & Sai, 2011; Zegers-Hochschild et al., 2009). Studies indicate that 50 to 80 million people worldwide experience some form of infertility during their life-time (Ramezanzadeh et al., 2007). So that, one couple out of six couples in childbearing age suffers from infertility in the world (Faal Kalkhoran et al., 2011). According to the World Health Organization, about 80 million people in the world are involved in pregnancy failure (Vayena & Organization, 2002). Some studies estimate the prevalence of infertility in the world between 10 to 15% (Jonaidy, Sadodin, Mokhber, & Shakeri, 2009; Kormi Nouri, 2000; Nikoubakht, Karimi, & Bahrami, 2011). Although some reports state its frequency in different parts of the world between 5 and 50 percent, the results of surveys of the prevalence of infertility in Iran report a prevalence of 12 to 21.9 percent (Fooladi et al., 2006). About one-fourth of Iranian couples experience primary infertility in their common life and 3.4 percent of couples suffer from primary infertility (Khodakarami, Hashemi, Seddigh, Hamdiyeh, & Taheripanah, 2010). In 40% of the cases, female factors; in 40% of the cases, male factors, and in 20% of the cases common factors have been reported to be responsible for infertility (Haririan, Mohammadpour, & Aghajanlou, 2010). Although infertility is not a disease but it can cause disorders in physical and mental health, quality of life, and impair the quality of marriage, and lead to separation and divorce, loss of self-confidence, feelings of grief, threatening, depression, guilt and frustration, emotional distress and bring about marital problems and rejection which in turn can lead to depression, anxiety or feeling of guilt (Haririan et al., 2010; Noorbala, Ramezanzadeh, Abedinia, & Naghizadeh, 2009). Several studies have referred to a negative impact of infertility on the quality of life in infertile women (Fekkes et al., 2003; Khayata, Rizk, Hasan, Ghazal-Aswad, & Asaad, 2003; Monga et al., 2004; Wilson JF, 2002). However, some studies have shown no difference between the overall quality of life of these people and that of the general population (Hearn, Yuzpe, Brown, & Casper, 1987; Weaver, Clifford, Hay, & Robinson, 1997). Some studies refer to sexual dysfunction as a consequence of infertility and state that during infertility 50 to 60 percent of couples had significantly lower sexual satisfaction (Sattarzadeh, 2004; Yektatalab Sh, 2004) and report factors such as low self-confidence, feelings of depression and anxiety, and sexual relationship overshadowed by the fear of failure to conceive as the cause for this (Bahrami, Sattarzadeh, Ranjbar Koochaksariie, & Ghojazadeh, 2007). Some studies report loss of libido, changes to the point of orgasm, decreased frequency of sexual intercourse and sexual dissatisfaction as problems of infertile couples, and due to these reasons, most of these people lose their interest in their lives, jobs and other things which were previously enjoyable activities for them, including sexual activities (Bahrami et al., 2007; Nikoubakht et al., 2011). Although some studies believe its effect is negligible, (Bahrami et al., 2007; Jonaidy et al., 2009; Kormi Nouri, 2000; Lee, Sun, & Chao, 2001), reduction in sexual satisfaction has many negative consequences such as crime, rape, divorce and psychological diseases (Kormi Nouri, 2000). Results of a study in Isfahan showed that there is a significant relationship between infertility and encouraging divorce, encourage remarriage, and adoption of a foster child (Shahnooshi & Karimi, 2010). Considering the importance of the issue and the lack of research in this area, this study intended to investigate the social implications of infertility for the families in Shahroud, a city in the North East of Iran.

## 2. Method

This study is a comparative study that was conducted in 2013. In this study, 1,528 participants (511 infertile and 1017 fertile ones) were randomly selected. To access15 to 49 year-old married infertile women in the rural population, the list of all infertile women was extracted from the notebook of continuous care and family planning, and twice as many participants were randomly selected from among 15 to 49 year-old married fertile women who had a history of at least one pregnancy. In the urban population, researchers referred to the obstetrics and gynecologists’ offices and after finding infertile 15 to 49 year-old married women, randomly also selected twice as many participants from married fertile women in the same age group. In practice, 301 infertile women in the rural areas and 210 infertile women the in urban areas were studied. The instrument used to determine the social consequences of infertility was Shahnooshy and colleagues’ questionnaire (Shahnooshi & Karimi, 2010), the validity and reliability of which was already confirmed (72%). The questionnaire includes 36 items, 18 of which are on the social consequences of infertility and 18 are about women’s attitudes towards infertility. After explaining the purpose of the study, questionnaires were self-administered. In the case of illiterate or low-literate subjects, the questionnaire was completed by a trained interviewer. To examine the relationship between variables, the chi-square test and one-way analysis of variance and t-test were used. Obtaining informed consent of the participants and their voluntary participation were the most important ethical considerations in the study. Also necessary permit for the study (Code 9103) were received from the Ethics Committee of the Shahroud University of Medical Sciences. In this study, confidence interval was 95% and the significance level was 0.05.

## 3. Findings

Due to census of all infertile women in the rural population (301 patients) and having the entire population of eligible women living in rural areas (13,471 people), the prevalence of infertility in rural areas was calculated to be 2.23 percent. 23.8% of the cases (n=364) had primary infertility, 9.6% (n=147) had secondary infertility, and 66.6% (n=1017) were fertile.

42.2% lived in the city (n=645) and 57.8% lived in the village (n=883). 84% of the cases under study were housewives (n=1287), and the rest were employed. 49.2% of the cases had education below high school diploma (n=751), 31.7% had high school diploma (n=484) and 19.2% had university degrees (n=293), in terms of spouse’s education, 51.1% had education below high school diplomas (n=781), 27.2% had diploma (n=415), 21.8% had education higher than diploma (n=332).

The income level of 27.1% of the cases was less than 100$ per month (n=414), 36.1% (n=552) had an income between 100-200$, 20.2% (n=309) had an income between 200-300$, and 16.5% (n=253) had an income over 300$.

51.9% of the participants referred to the infertility problem among distant relatives, 24.9% referred to infertility among close relatives and 9% reported the infertility among their family members. The greatest cause of disability in fertility was related to the individual herself (34.7%), the 19.5% was related to husband, and 22.5% was related to both wife and husband, and 23.4 percent was unexplained. In terms of the kinship between the participants and their husbands, results showed that 52.9% were marriages with non-relatives, 21% with distant relatives and the remaining (26.1%) were with close relatives. 80% of participants had applied for the treatment of infertility.

The most frequent reason of inaction for treatment was financial problems and lack of cooperation of the spouse (75.7%), and then was lack of access to reliable infertility treatment centers (10.3%). 39.3% of infertile women referred to the interference of family members in their lives due to their infertility. 20.4% of infertile women accused their husband of infertility and 43.6% of infertile women felt their wishes were destroyed.

Chi-square test showed a significant difference between regularity of menstruation and fertility status so that fertile women had more regular menstrual cycles than infertile (P=0.001, X^2^=50.897). Chi-square test showed no significant difference between the problem of infertility in relatives and infertility (P=0.163, X^2^=1.943) (Tables 1 and 2).

**Table 1:** The relationship of fertility status and some variables

Variable		Fertility status		x^2^	P

		Infertile	Fertile		
Menstruation	regular	303(60.4)	781(77.9)	50.897	0.001
	irregular	199(39.6)	222(22.1)		
Ability to conceive a child	Yes	160(33.1)	904(91.1)	5.452	0.001
	No	324(66.9)	88(8.9)		
Infertility among relatives	Yes	153(30.8)	329(34.4)	1.943	0.163
	No	344(69.2)	627(65.6)		

**Table 2:** Comparison of mean of variables in fertile and infertile women

Variable	Mean ± SD		t	P

	Infertile	Fertile		
Age at menarche	13.37±1.65	13.50±1.60	-1.40	0.154
Duration of marriage (years)	9.64±7.34	11.16±7.27	-3.79	0.001
Age	31.04±7.39	31.43±6.80	-0.999	0.318
Spouse’s age	35.86±9.37	35.97±7.48	-0.230	0.818
Age at first marriage	21.08±5.31	20.18±4.24	-3.322	0.001
Attitude	56.60±7.03	56.81±6.66	-0.567	0.571

**Table 3:** Comparison of interventions made by the relatives of the two groups

Type of intervention		Fertility status		x^2^	P

		Fertile	Infertile		
		N (%)	N (%)		
Encouraging divorce	No	1012(67.2)	494(32.8)	19.27	0.001
	Yes	5(22.7)	17(77.3)		
Encouraging remarriage	No	1012(67.9)	478(32.1)	49.92	0.001
	Yes	5(13.2)	33(86.8)		
Encouraging adoption	No	1014(68.5)	466(31.5)	80.98	0.001
	Yes	3(6.3)	45(93.8)		

Chi-square test showed a significant relationship between fertility status and encouraging divorce, remarriage and encouraging adoption so that interventions in infertile women group was significantly higher than that in fertile women group.

## 4. Discussion

The prevalence of infertility in rural areas in this study was 2.23 percent. In a study entitled “Study of Women Infertility in Gonabad” the prevalence of infertility in rural areas was 11.4 percent (Sadegh Moghadam, Moslem, Gharche, & Chamanzari, 2008). In a study, the researchers announced the prevalence of infertility in 20-49 year-old women in Tehran to be 21.9 percent (Barooti, Ramezani Tehrani, Heydari Seraj, & Ashrafi, 1999) and in another study the prevalence of infertility in the West of Tehran was reported 12 percent (Nojoomi, 2001). The higher prevalence of infertility in all these studies is inconsistent with the current study. It seems that certain factors which require further examination have lowered the infertility index compared to other studies. Our estimate is for rural area and with respect our sampling method in urban area we cannot present a valid estimation for prevalence.

The attitude of participants toward infertility did not show significant differences in fertile and infertile individuals. In a study entitled “Attitude towards Infertility and Its Relation to Depression and Anxiety in Infertile Couples” did not find a relationship between infertility and attitudes (Nilforooshan, Ahmadi, Abedi, & Ahmadi, 2006). In another study, the researchers found no difference between the attitudes of men and women (Fooladi et al., 2006), which is consistent with recent results.

In our study, there was a significant relationship between the length of the marriage and infertility. Shahnooshi M. and colleagues in a study entitled “the Sociological Impact of Infertility upon Family in Isfahan” found a relationship between duration of marriage and infertility (Shahnooshi & Karimi, 2010), which is consistent with recent results. It seems that with the passage of time, the probability of fertility increases and people can get a better outcome than those who look for hasty treatment in the early stages of their marriage.

A significant relationship was observed between infertility and encouraging divorce, which is consistent that the results of Isfahan study (Shahnooshi & Karimi, 2010). Existence of infertility in both men and women and interventions made by families and relatives to have a life with children, encourage each of the couple to divorce.

A significant relationship was also observed between infertility and encouraging remarriage which is consistent with the findings of Shahnooshi M. and colleagues (Shahnooshi & Karimi, 2010). Perhaps the desire of couples and others’ encouragement, and a sense of family survival in the event of remarriage are the reasons for this.

A further significant relationship was also observed between infertility and the adoption of foster child which is again consistent with the results of Isfahan study (Shahnooshi & Karimi, 2010). In fact, this indicates that infertile families with such a reasonable choice of adopting a child prevent many of the outcomes of infertility.

Significant relationships were also observed between infertility and a number of factors including the length of the marriage, age of marriage, regularity of menstrual periods. In his study, Sadegh Moghaddam L. reported no significant relationship between the individual characteristics of women, their menstrual cycle and infertility (Sadegh Moghadam et al., 2008) which is not consistent with the results of our study.

## 5. Conclusion

Infertility does not cause a negative attitude toward infertile people. However, the intervention of others, and encouraging divorce and remarriage are higher among infertile people. According to the fact that about half of those considered infertile feel their wishes are destroyed, attention to midwifery and mental health counseling of this group seems necessary.
